# Prebiotic aqueous reactions catalyzed by native nickel without hydrogen

**DOI:** 10.1111/febs.70556

**Published:** 2026-04-18

**Authors:** Carolina Garcia Garcia, Max Brabender, William F. Martin

**Affiliations:** ^1^ Institute of Molecular Evolution Heinrich Heine University Düsseldorf Germany

**Keywords:** hydrogen, hydrothermal vents, native metals, origin of life, origin of metabolism, serpentinization

## Abstract

Compared to iron, nickel is comparatively rare as a transition metal in enzymes. However, it is essential in several enzymes of carbon and energy metabolism in acetogens (bacteria) and methanogens (archaea), which use the acetyl‐CoA pathway of H_2_‐dependent CO_2_ fixation. Nickel containing enzymes of acetogens and methanogens include FeNi hydrogenase, carbon monoxide dehydrogenase, acetyl‐CoA synthase and, in methanogens, methyl‐CoM reductase in the last step of methane synthesis. Several lines of evidence implicate the acetyl‐CoA pathway as the most ancient pathway of CO_2_ fixation, most notably recent findings that the overall reaction of the enzymatic pathway from H_2_ (*E*_0_′ = −414 mV) and CO_2_ to pyruvate can be replaced by Ni^0^ alone in water as the lone catalyst. Here, we studied Ni^0^ as a catalyst and reductant for nonenzymatic redox reactions that require only a mild reductant, as the midpoint potential of Ni^0^ oxidation to Ni^2+^ is *E*_0_′ = −260 mV. We showed that Ni^0^ in water can convert 2‐oxo acids to 2‐hydroxy acids and, in the presence of NH_3_, to amino acids at 25–100 °C without the addition of H_2_, and that it functions as a catalyst and reductant for the fumarate reductase reaction. The findings expand the repertoire of ancient metabolic reactions that Ni^0^ can catalyze without proteins, cofactors, or sulfur, shedding light on the broad catalytic activity and substrate specificity of Ni^0^ at metabolic origin.

## Graphical abstract

Serpentinizing (H_2_‐producing) hydrothermal vents are candidate environments for metabolic origin. They generate highly reducing conditions that convert CO_2_ to formate and methane in abiotic reactions resembling reactions of the acetyl‐CoA pathway of CO_2_ fixation. They also contain natural catalysts. Native nickel (Ni^0^), like Fe^0^, Co^0^, and their alloys, is naturally deposited in serpentinizing vents. Nickel is a broad‐specificity catalyst of biochemical reactions at the origin of metabolism and is conserved in ancient enzymes and cofactors.

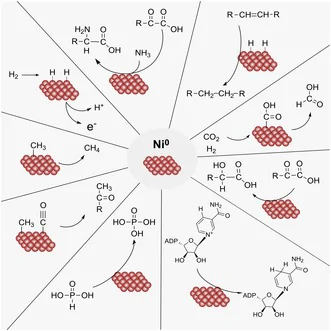

**Abbreviations**
ACS: acetyl‐CoA synthase
CODH: carbon monoxide dehydrogenase
Hyd: hydrogenases
MCR: methyl‐CoM reductase
NPN: nickel‐pincer nucleotide

## Introduction

Metabolism is a chemical reaction. It emerged from reactions catalyzed by environments present on the early Earth and gave rise to the metabolic reaction networks of living cells [1, 2, 3, 4]. Modern microbes possess enzymes and cofactors that accelerate the reactions of metabolism so that all reactions take place at roughly the same rate [5]. At the very onset of metabolism, there were no enzymes or cofactors, only inorganic catalysts [6]. Transition metals play a crucial role in theories for metabolic origin because they are notoriously good at accelerating particularly difficult chemical reactions such as N_2_ reduction [7]. Coordinated in proteins and cofactors, Fe, Ni, Co, and Mo (sometimes replaced by W [8]) are essential to catalysis in anaerobic autotrophs and are abundant in enzymes of the acetyl‐CoA pathway [9, 10, 11, 12, 13, 14]. Among pathways of CO_2_ fixation, the acetyl‐CoA pathway is the most ancient, the only one that occurs in bacteria and archaea [15, 16] and the only one that serves both carbon and energy metabolism [13, 17]. It is the starting point of metabolism in acetogens (bacteria) and methanogens (archaea) respectively [17, 18], and it traces to the last universal common ancestor [19, 20].

The most common transition metal in enzymes is Fe, which usually occurs in electron‐transferring FeS clusters [21]. As an example, formyl‐methanofuran dehydrogenase of the acetyl‐CoA pathway in methanogens contains 46 electron‐transferring 4Fe4S clusters [22]. By contrast, Ni is rarely required by enzymes, and when it occurs, it participates in catalysis at the active site (reviewed by [23, 24]). It is found in the active site of (i) urease—the first protein ever crystallized [25]—of (ii) glyoxylase in methylglyoxal detoxification [26], of (iii) acireductone dioxygenase in the methionine salvage pathway [27], and of (iv) Ni‐superoxide dismutase [28, 29, 30], and in the active site of three enzymes of the acetyl‐CoA pathway: In [Fe‐Ni] hydrogenases (Hyd), Ni catalyzes the H_2_‐dependent reduction of ferredoxin [31, 32, 33, 34, 35, 36]. In carbon monoxide dehydrogenase (CODH), Ni catalyzes the reduction of CO_2_ to CO [14, 34, 37, 38, 39, 40, 41]. In acetyl‐CoA synthase (ACS), Ni catalyzes the synthesis of acetyl‐CoA from CO, CoASH and a methyl group [14, 34, 39, 41, 42]. In methanogens, nickel in F_430_ also catalyzes the final step of methane synthesis at methyl‐CoM reductase (MCR) [43, 44, 45, 46, 47]. The antiquity CO_2_ fixation via the acetyl‐CoA pathway [13, 17] and the 3.5 Ga age of methanogenesis [48] trace Ni‐based catalysis to the onset of biochemical evolution [49].

Although Ni in modern enzymes and cofactors is typically coordinated by sulfur or nitrogen atoms, [24, 50] this need not represent the ancestral state of catalytic Ni at origins. Inorganic NiS complexes can catalyze the ACS reaction starting from CO and methyl groups [51]. The reduction of CO_2_ using NiS or FeS does not, however, take place unless external potentials of ~1000 mV are applied [52, 53, 54], whereby that catalysis is afforded not by metal sulfides, but by native metals that are formed from the sulfides on the electrode during the electrochemical reaction [55]. Without electrodes, pure Ni^0^ is an excellent catalyst of CO_2_ reduction. In the absence of enzymes, Ni^0^ in water catalyzes the H_2_‐dependent reduction of CO_2_ to the products of the acetyl‐CoA pathway—formate, acetate, and pyruvate—in hours to days at 50–100 °C [56]. That is, Ni^0^ nanoparticles replace the function of 10 enzymes and 10 cofactors of the acetyl‐CoA pathway, which require the activity of 127 enzymes in cells [57]. FeNi alloys and FeCo alloys as well as Fe^0^ alone catalyze the same spectrum of reactions as Ni^0^ [58, 59, 60, 61, 62, 63]. Beyond the reactions of the acetyl‐CoA pathway, Ni^0^ catalyzes reactions of the reverse TCA cycle and H_2_‐dependent reductive aminations of various 2‐oxoacids to amino acids [64]. Ni^0^ will catalyze the reduction of NADH (*E*_0_′ = −320 mV) with H_2_ (*E*_0_′ = −414 mV) [65, 66], and it will reductively aminate pyridoxal to pyridoxamine using H_2_ [67], though it will not reduce low potential ferredoxin (*E*_0_′ = −450 mV) with H_2_, whereas Fe^0^ (*E*_0_ = −440 mV at pH0; *E*_0_ ca. –800 mV) at pH14 [68] will [69].

Native nickel and its alloys are naturally deposited in serpentinizing hydrothermal vents by reduction of divalent metal ions with H_2_ generated during the serpentinization process [70, 71, 72]. Nickel alloys are implicated in the purely geochemical synthesis of methane [73] that occurs in modern serpentinizing hydrothermal systems [74]. Given that serpentinization has been going on since there was liquid water on Earth [3], nickel‐based CO_2_ reduction and aminations likely operated before enzymes ever existed and continued to operate during the earliest phases of biochemical evolution as the first enzymes and pathways were arising [64, 66, 67, 69, 75, 76].

While the utility of Ni in enzymes lies in its ability to readily undergo changes of valence state [9, 77], its utility in catalysis of H_2_‐dependent reductions of organic compounds lies in its ability to absorb and activate H_2_—a property that has been exploited in organic chemistry for over 100 years [78]. Nickel particles avidly bind H_2_ as nickel hydride, Ni–H, at particle surfaces. At 1 atm H_2_, essentially all Ni atoms exposed at the surface of a Ni^0^ nanoparticle are occupied as Ni–H hydride [79]. At higher H_2_ partial pressures, H diffuses into deeper atom layers as well, and at 30 atm of H_2_, a 2.7 nm diameter Ni nanoparticle can bind up to 3% H_2_ by weight [78]. For comparison to natural and biological systems, the effluent of serpentinizing hydrothermal vents contains 1–10 atm H_2_ [80], methanogens require only 10^−5^–10^−4^ atm H_2_ to grow [81], while acetogens require 6·10^−5^–10^−3^ atm H_2_, depending on the strain [82]. Ni–H formation from Ni^0^ and H_2_ is both spontaneous and facile [79], yet it is not a redox reaction, as both Ni and H remain in their elemental state. Like Co [83, 84], Mo [85, 86], and Fe [87, 88] nickel also occurs in cofactors: the Ni‐tetrapyrrole F_430_ of methanogens and in the nickel‐pincer nucleotide (NPN) cofactor [89] first described in lactate racemase [90].

The midpoint potential of Ni^0^ oxidation to Ni^2+^ under physiological conditions is *E*_0_′ = −260 mV, sufficient to reduce a number of common biological substrates, for example pyruvate to lactate (*E*_0_′ = −190 mV) in the absence of H_2_. With rare exceptions [67], the use of Ni^0^ in experimental reconstructions of metabolic origin so far has focused on H_2_‐dependent reductions. Here we investigate reactions relevant to prebiotic chemistry using Ni^0^ as catalyst and reductant.

## Results

In the presence of H_2_, nickel supplied as commercial nickel silicate powder (Ni‐SiO_2_/Al_2_O_3_) catalyzes the reduction of pyruvate to lactate and, when ammonium is added redundant, pyruvate is reductively aminated to alanine [64, 67]. However, the same reactions take place, though to a lesser extent, in the absence of H_2_. We incubated 20 mm pyruvate with and without 20 mm ammonium chloride in the presence or absence of Ni‐SiO_2_/Al_2_O_3_ catalyst under 5 bar Ar. After 18 h at 100 °C and pH 11, a pH typical of serpentinizing hydrothermal systems [80, 91], 42% of pyruvate was reduced to lactate in the presence of Ni‐SiO_2_/Al_2_O_3_ and 6.4% of pyruvate was reductively aminated to alanine in the presence of Ni‐SiO_2_/Al_2_O_3_ and ammonia (Fig. 1; Table S1). Without the catalyst, no alanine or lactate accumulates. When Ni^0^ is present but ammonia is absent, only lactate is observed at a conversion rate of 49% (Fig. 1, Table S1).

**Fig. 1:**
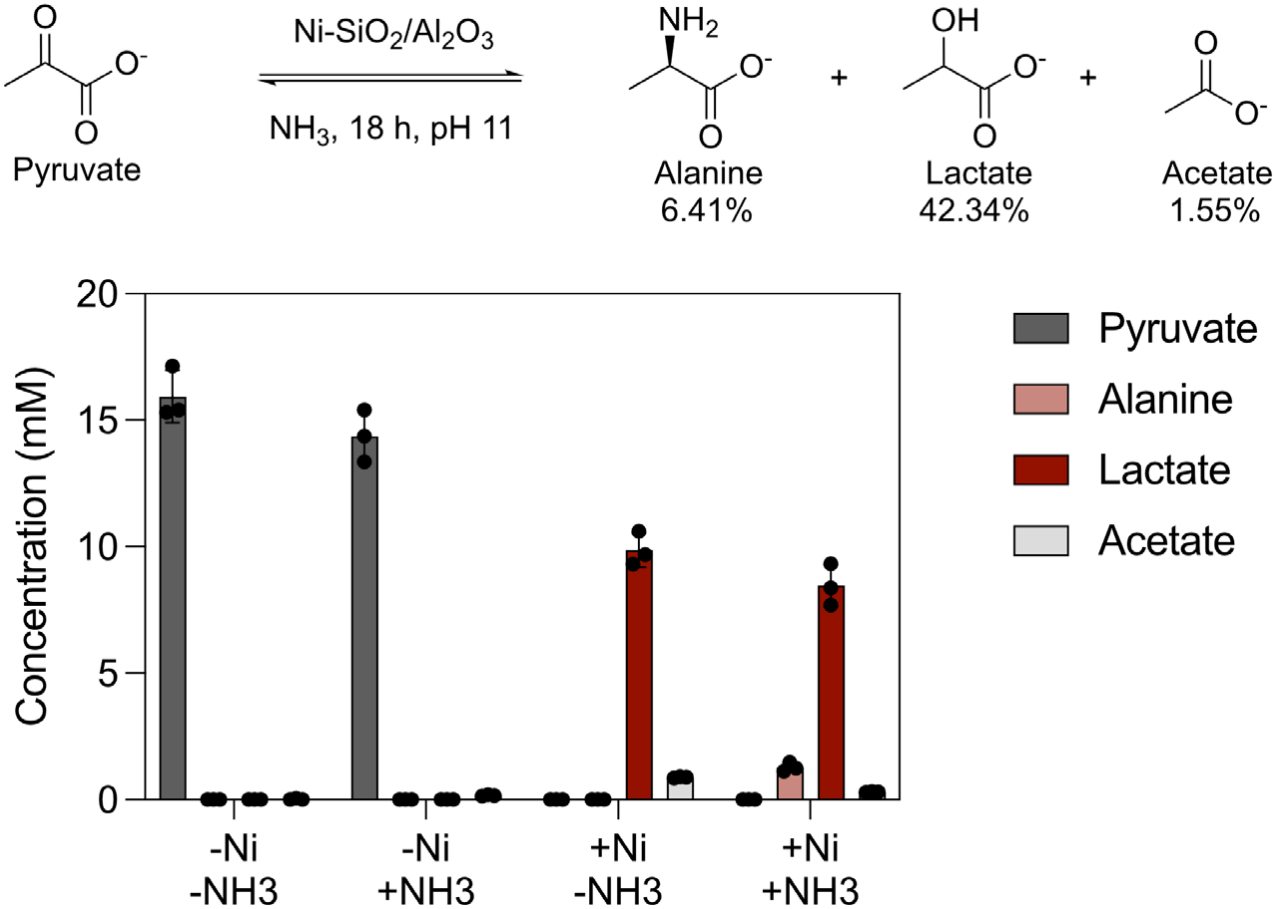
Alanine synthesis in the presence of nickel catalyst. Initial concentrations were 20 mm pyruvate and 200 mm ammonium chloride. Ni‐SiO_2_/Al_2_O_3_ (1 mmol of Ni atoms) was added as solid phase powder in a total reaction volume of 1.5 mL. The reaction was performed under a 5 bar Ar atmosphere, initial pH 11 with KOH; the reaction time was 18 h at 100 °C. No H_2_ was added. Error bars in the figure indicate standard deviation (SD). Reactions were performed in triplicates.

This suggested that under the conditions of actively serpentinizing vents, nickel (*E*_0_′ = −260 mV), present as 1 mmol metal atoms versus 30 mmol pyruvate in the reaction, is acting not only as a catalyst but also as an electron donor for lactate synthesis (*E*_0_′ = −190 mV) as well. However, pyruvate could also be undergoing oxidation during the reaction and thus could, in principle, also serve as an electron donor. To test this, we examined parameters impacting the reduction of pyruvate to lactate.

First we examined the effect of pH. In actively serpentinizing hydrothermal systems, pH can typically vary in a range of 8–10, reaching pH 12 or higher in hyperalkaline systems (Cedars, [92]; Oman ophiolite, [93]; Hakuba happo, [94]). After 2 h at 100 °C at pH 7–11, we observed 86% conversion of pyruvate to lactate at pH 9 (Fig. 2; Table S2), such that pyruvate cannot be serving as the (sole) electron source via pyruvate disproportionation: pyruvate oxidation to CO_2_, acetate, and H_2_, the latter reducing pyruvate over Ni^0^ to lactate. Acetate formation was very low, and increases with pH, whereas lactate peaks at pH 9.

**Fig. 2:**
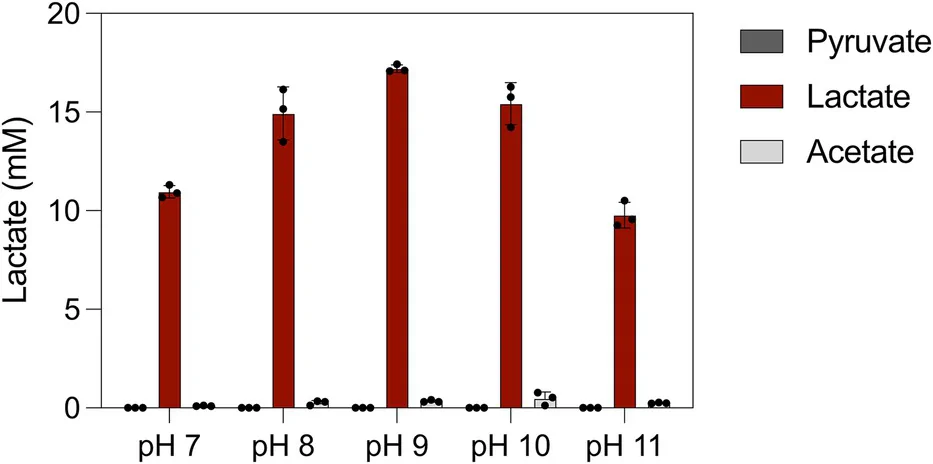
Effect of pH on lactate synthesis in the presence of nickel catalyst. Initial concentrations were 20 mm of pyruvate. Ni‐SiO_2_/Al_2_O_3_ (1 mmol of Ni atoms) was added as solid phase powder in a total reaction volume of 1.5 mL. The pH was set to 7, 8, 9, 10, and 11 with KOH, respectively, and the reaction time was set to 2 h. The reaction was performed under a 5 bar Ar atmosphere. No H_2_ was added. Each dot represents a single measurement. Dots positioned on the X‐axis represent measurements where no concentration could be detected. Error bars in the figure indicate standard deviation (SD). Reactions were performed in triplicates.

Temperature gradients exist in serpentinizing hydrothermal vents. Biological reactions in the absence of an enzyme generally proceed more rapidly at increased temperature [5]. Pyruvate reduction did not proceed at temperatures below 60 °C (Fig. 3A–D; Table S3) and was essentially complete after 2 h (Fig. 3B; Table S3) at 80–100 °C with 82–86% conversion. In 18 h reactions (Fig. 3D; Table S3), lactate decreases, likely due to thermal sensitivity [95].

**Fig. 3:**
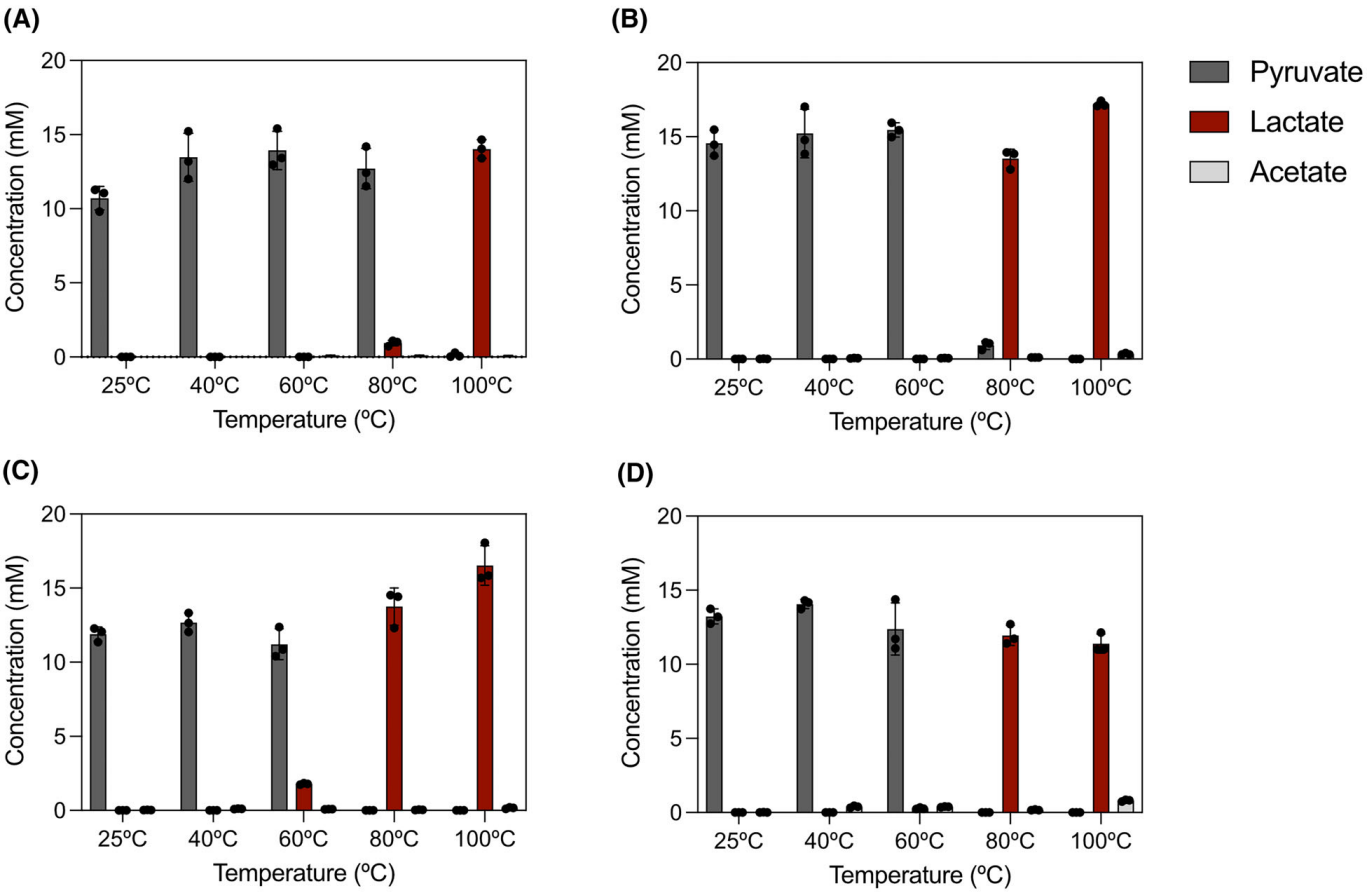
Effect of temperature and time on lactate synthesis in the presence of nickel catalyst. Initial concentrations were 20 mm of pyruvate. Ni‐SiO_2_/Al_2_O_3_ (1 mmol of Ni atoms) was added as solid phase powder in a total reaction volume of 1.5 mL. The reaction time was 1 h (A), 2 h (B), 4 h (C), and 18 h (D), respectively. Temperature was 25, 40, 60, 80, and 100 °C. The pH was set to 9 with KOH. The reaction was performed under a 5 bar Ar atmosphere. No H_2_ was added. Error bars in the figure indicate standard deviation (SD). Reactions were performed in triplicates.

Using commercial nano nickel powder and micro nickel powder, no reduction was observed after 2h (Fig. 4A; Table S4), and only partial reduction to lactate was observed after 18 h (Fig. 4B; Table S4) with a yield of 37% with nano nickel powder and 15.9% with micro nickel powder. The silica matrix itself does not reduce pyruvate (Fig. 4A‐B; Table S4).

**Fig. 4:**
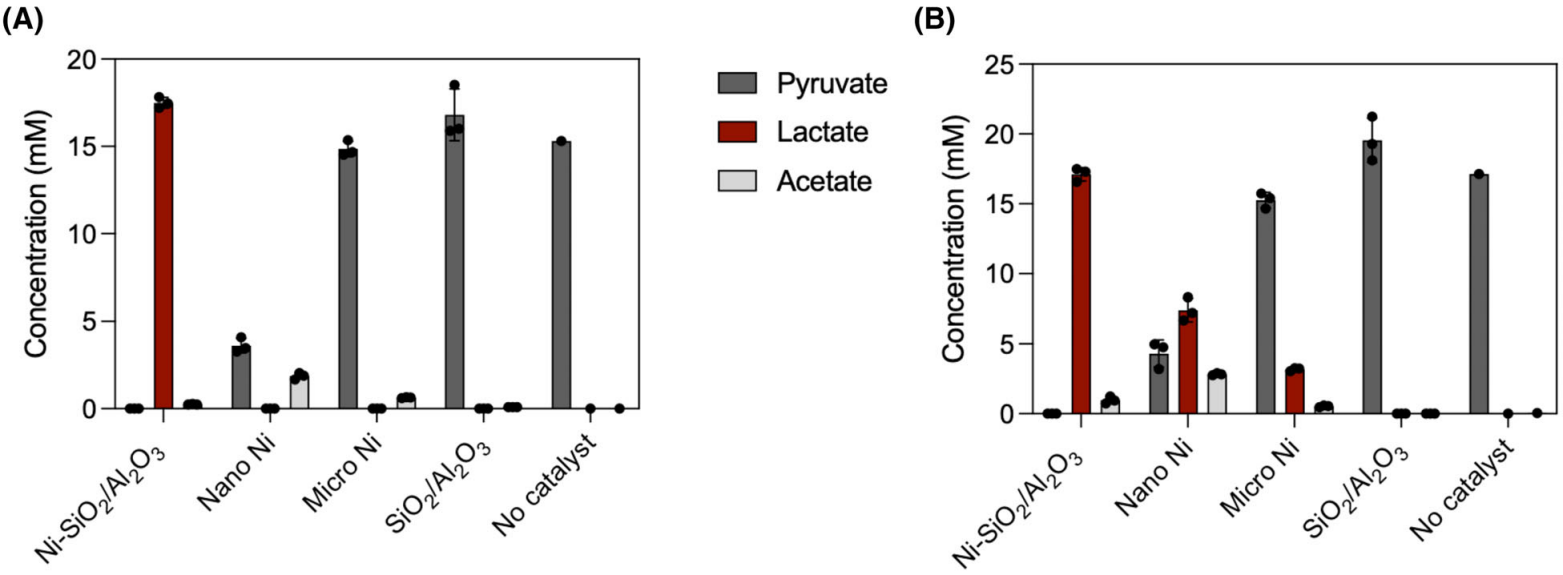
Effect of different catalysts on lactate synthesis. Pyruvate concentration was set to 20 mm. The catalysts (Ni‐SiO_2_/Al_2_O_3_, Nano nickel powder, micro nickel powder, SiO_2_/Al_2_O_3_) were added as 1 mmol of Ni atoms of undissolved solid phase powder in a total reaction volume of 1.5 mL. The reaction was performed at 100°C under a 5 bar argon atmosphere; pH was set to 9 with KOH. No H_2_ was added. The reaction time was set to 2 h (A) and 18 h (B). Error bars in the figure represent standard deviation (SD). Each reaction was performed in triplicates.

Pyruvate was reduced to lactate using as little as 0.05 mmol of Ni per reaction, and the yield steadily increases to 0.66 mmol of Ni per reaction. The yield plateaus at 1 mmol of Ni as Ni‐SiO_2_/Al_2_O_3_ with 86% conversion (Fig. 5; Table S5).

**Fig. 5:**
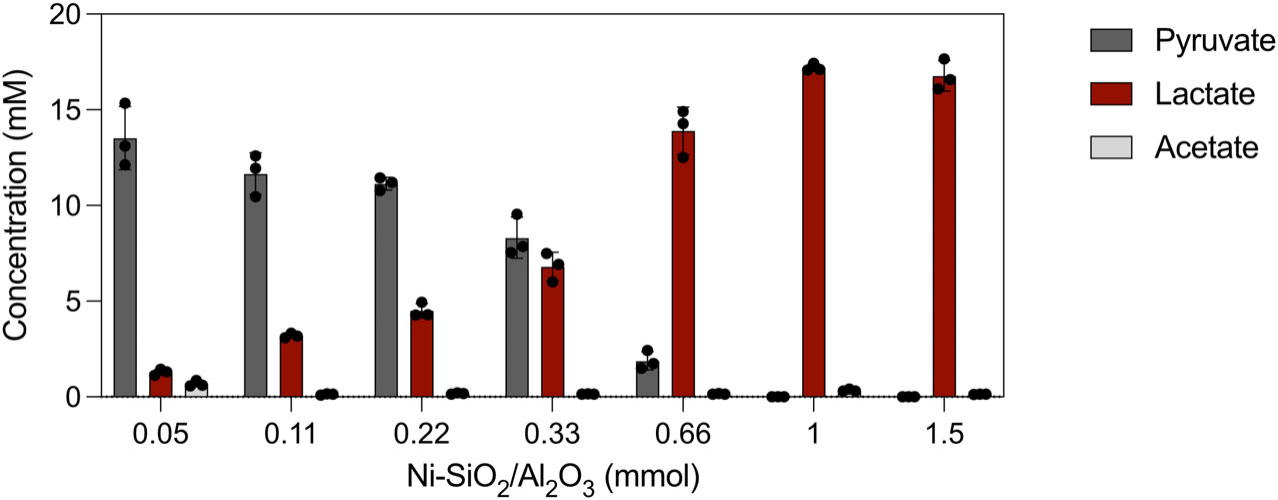
Effect of catalyst concentration on lactate synthesis in the presence of nickel catalyst. Initial concentrations were 20 mm of pyruvate. Ni‐SiO_2_/Al_2_O_3_ was added as solid phase at a concentration of 0.05, 0.11, 0.22, 0.33, 0.66, 1, and 1.5 mmol of Ni atoms, respectively. The pH was 9 (KOH), and the reaction was performed for 2 h at 100°C, under a 5 bar Ar atmosphere. No H_2_ was added. Error bars in the figure represent standard deviation (SD). Each reaction was performed in triplicates.

Kaur *et al*. (2024) converted 2‐oxoglutarate, 4‐methyl‐2‐oxopentaonate, and 3‐methyl‐2‐oxopentaonate into the corresponding amino acids using Ni/H_2_ and NH_3_. We reacted 20 mm 2‐oxoacid and 1.5 mm Ni as Ni‐SiO_2_/Al_2_O_3_ under 5 bar Ar at pH 9 and 100 °C. We also tested the possibility of nickel performing double bond reductions. After 2 h at pH 9, fumarate was reduced to succinate (*E*_0_′ = −130 mV) with a 100% conversion rate (Fig. 6A; Table S6) The reduction of 2‐oxoglutarate to 2‐hydroxyglutarate (*E*_0_′ = −337 mV) had a 100% conversion, the reduction of 4‐methyl‐2‐oxopentanoate to hydroxyisocaproate (*E*_0_′ = −344 mV) was 97% complete after 2 h, while the reduction of 3‐methyl‐2‐oxopentanoate to 2‐hydroxy‐3‐methylvalerate (*E*_0_′ = −357 mV) underwent 64% conversion (Fig. 6B–D; Table S6).

**Fig. 6:**
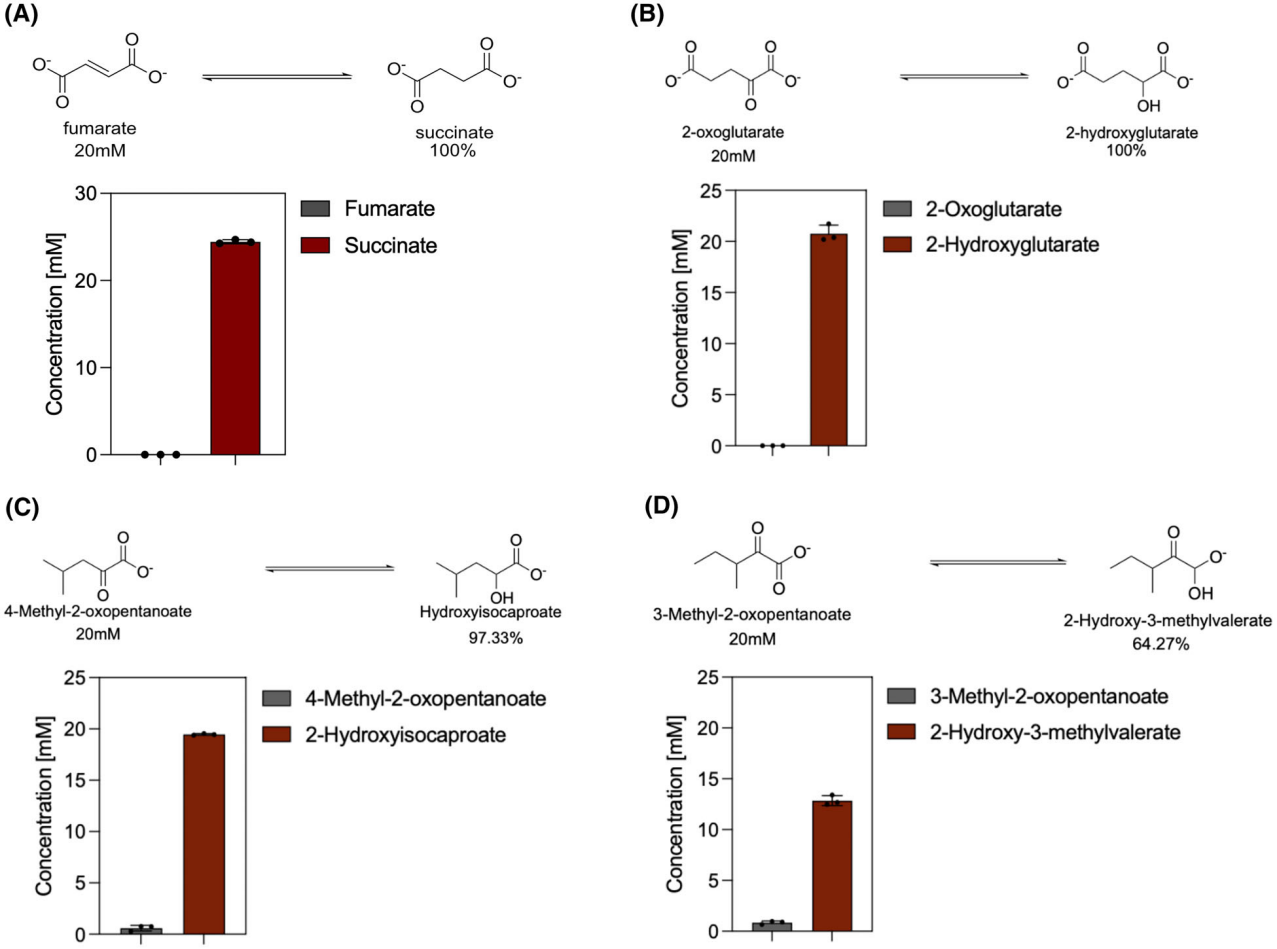
Ketone and double bond reduction in the presence of nickel catalyst. Educt concentration was set to 20 mm. Ni‐SiO_2_/Al_2_O_3_ (1 mmol of Ni atoms) was added as solid phase powder in a total reaction volume of 1.5 mL. The reaction time was set to 2 h at 100 °C, and pH was set to 9 with KOH. The reaction was performed under a 5 bar Ar atmosphere. No H_2_ was added. Error bars in the figure represent standard deviation (SD). Each reaction was performed in triplicates. (A) Succinate synthesis from fumarate. (B) 2‐Hydroxyglutarate synthesis from 2‐oxoglutarate. (C) 2‐Hydroxyisocaproate synthesis from 4‐methyl‐2‐oxopentanoate. (D) 2‐Hydroxy‐3‐methylvalerate synthesis from 3‐methyl‐2‐oxopentanoate.

We investigated the reductive amination of these alpha‐ketoacids to their corresponding amino acids using ammonium, with nickel as the reductant, adjusting the length of the reaction time (72 h) and the pH (pH 11) to improve yields. Under these conditions, nickel has a lower midpoint potential [96], allowing for the reductive amination of the respective compounds. We observed that 4‐methyl‐2‐oxopentanoate underwent reductive amination to leucine (*E*_0_′ = −386 mV) with 6.6% conversion rate and 3‐methyl‐2‐oxopentanoate was reductively aminated to isoleucine (*E*_0_′ = −419 mV) with a 4.8% conversion rate (Fig. 7A,B; Table S7). Reductive amination of 2‐oxoglutarate did not generate glutamate (*E*_0_′ = −380 mV) but its cyclic peptide derivative 5‐oxoproline, which is known to occur at high temperature and high pH [97]. The formation of 5‐oxoproline indicates reductive amination to 2‐oxoglutarate at 57% (Fig. 7C; Table S7).

**Fig. 7:**
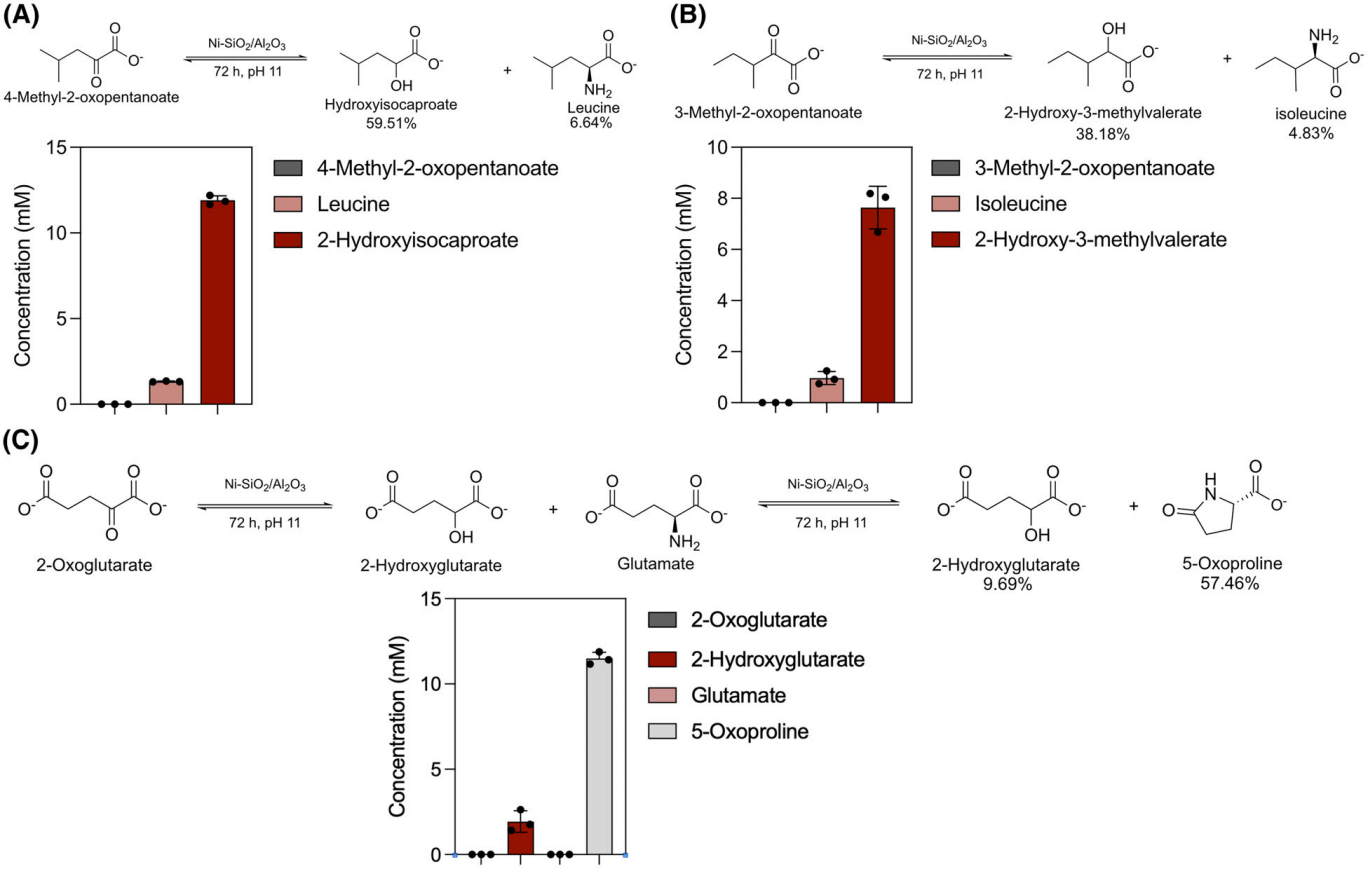
Reductive amination in the presence of nickel catalyst. Initial concentrations were 20 mm of each respective educt and 200 mm ammonium chloride. Ni‐SiO_2_/Al_2_O_3_ (1 mmol of Ni atoms) was added as solid phase powder in a total reaction volume of 1.5 mL. The reaction was performed under a 5 bar Ar atmosphere, initial pH 11 with KOH, the reaction was set at 72 h and 100 °C. No H_2_ was added. Error bars in the figure indicate standard deviation (SD). Reactions were performed in triplicates. (A) Leucine synthesis from 4‐methyl‐2‐oxopentanoate. (B) Isoleucine synthesis from 3‐methyl‐2‐oxopentanoate. (C) 5‐Oxoproline synthesis from 2‐oxoglutarate.

## Discussion

Serpentinizing hydrothermal systems are interesting sites for the origin of metabolism because they generate a constant supply of H_2_ for CO_2_ reduction [49, 98, 99] and because there is broad congruence between reactions catalyzed under hydrothermal conditions and reactions of metabolism [19, 97]. The process of serpentinization furthermore generates highly alkaline effluent, pH 9–12, producing strongly reducing conditions with potentials on the order of −800 mV or more [90], which are sufficient to reduce divalent metals to native metals [70, 72, 100, 101, 102, 103]. Native metals deposited in serpentinizing hydrothermal vents include Fe, Co, Ni, Pd, other platinum group elements (PGE) and their alloys [70, 103, 104, 105]. These metals activate H_2_ via chemisorbtion and catalyze organic reactions [60, 61, 62, 63] under conditions conducive to the origin of metabolism. A number of recent studies have shown that Ni^0^ can promote the conversion of CO_2_ to organic acids using H_2_ as the reductant [60, 62, 63], retracing rather exactly the reactions of the acetyl‐CoA pathway [60, 62, 63] and reactions of the reverse TCA cycle [58, 64, 106]. In water, Ni^0^, Fe^0^ and Co^0^ are furthermore compatible with cofactors, catalyzing the H_2_ dependent reduction of NAD^+^ [65, 66], while Ni^0^ efficiently catalyzes the H_2_ dependent reduction of pyridoxal [67], and Fe^0^ catalyzes the reduction of 4Fe4S clusters in ferredoxin [69]. With a midpoint potential of 440 mV, Fe^0^ can, and does, generate H_2_ in water, and can reduce CO_2_ without additional reductants [60]. But Ni^0^ (*E*_0_′ = −270 mV) is a milder reductant.

Here we have shown that under serpentinizing conditions (high pH, temperature 50–100 °C) Ni^0^ can serve as a catalyst and reductant for reduction of 2‐oxo groups to hydroxyl, reduction of double bonds, and reductive amination of 2‐oxo acids to amino acids. These findings underscore the broad compatibility of native Ni with metabolic reactions. In modern metabolism, Ni is usually coordinated by S, C and N ligands in enzymes and cofactors as the divalent ion, but can undergo valence state changes during enzymatic reactions [23, 24, 45]. The extremely broad range of biochemical reactions that Ni^0^ can catalyze with or without H_2_ as a reductant and their similarity, often identical, to the reactions of metabolism in terms of reactants and products indicate that Ni^0^ was involved in metabolic origin, as outlined in Fig. 8A–I.

**Fig. 8:**
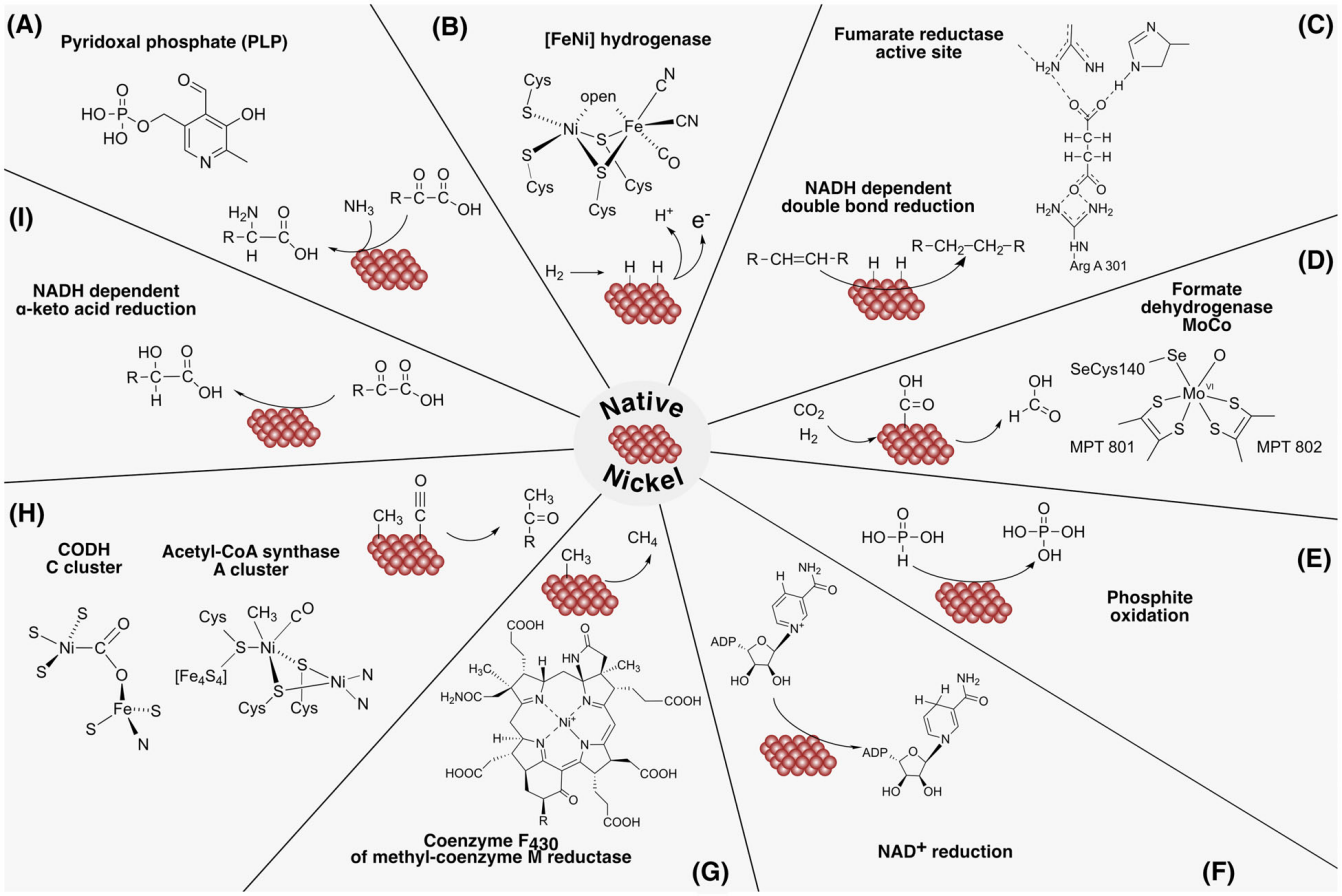
Broad substrate specificity of nickel as a primordial catalyst in metabolism. (A) Reductive amination: the presence of pyridoxal improves the yield [67], but the reaction also proceeds in the presence of Ni^0^ alone (this paper). (B) H_2_ oxidation: structure of carbon‐Ni bond present in [FeNi] hydrogenase [31, 32]. Ni^0^ activates H_2_ [60] (C) Double bond reduction: active site of fumarase taken from [107], the presence of Ni^0^ alone catalyzes the reaction (this paper). (D) CO_2_ fixation: structure of molybdenum cofactor of formate dehydrogenase [108]; Ni^0^ catalyzes the reaction under hydrothermal vent conditions [60]. (E) Phosphite oxidation [97]. (F) NAD^+^ reduction [65, 66]. (G) Methane formation: Ni^0^ generates methane from H_2_ and CO_2_ [76], the biological reaction requires coenzyme F_430_ in methyl‐coenzyme M reductase [43]. (H) CO_2_ fixation: structure of the carbon‐Ni bonds present in carbon monoxide dehydrogenase [37, 38, 39, 40], and acetyl‐CoA synthase A‐cluster [39, 40, 41, 42]; Ni^0^ catalyzes acetyl synthesis under hydrothermal vent conditions [60]. (I) α‐keto acid reduction: catalyzed by nickel (this paper).

Yet if Ni^0^ is so effective as a catalyst, one might ask why is Ni^0^ not used as a catalyst by enzymes or cofactors in modern metabolism. Cells can easily generate the potential needed to convert Ni^2+^ to Ni^0^, with the help of low potential reduced ferredoxin (*E*_o_ = −450 mV) [109] or using H_2_ with electron bifurcation [110]. Why is Ni^0^ not used by metabolism in cells?

The answer, we suggest, is specificity. Broad substrate specificity is a classical trait of ancient enzymes [111, 112]. The first enzymes likely had very broad substrate specificity for a given kind of reaction (reductive amination for example) and diversified into ancient enzyme families each with greater substrate specificity, so that the reactions of (proto‐) metabolism proceeded along orderly and well‐defined lines. Native nickel is a highly effective catalyst, but catalyzes *too many* different kinds of reactions (Fig. 8A–I). Ni^0^ catalyzes H_2_ activation (Fig. 8B) [60, 62, 67] as in hydrogenases [32, 33, 34, 35, 36] and CO_2_ reduction (Fig. 8D) to formate, acetate, and pyruvate [60, 62] including the formation of C–C bonds [60, 62, 63, 64] as in the acetyl‐CoA pathway (Fig. 8H) [17, 60]. It catalyzes reactions of the rTCA cycle [58, 64, 106]. And, as shown here, Ni^0^ catalyzes keto to alcohol reductions (Fig. 8I), reductive transaminations (Fig. 8A), and the reduction of double bonds in the absence of additional reductants (Fig. 8C). All of those reactions take place in water across a broad neutral to alkaline pH range and a wide range of biologically relevant temperatures. The extremely broad substrate specificity of Ni^0^ would make it an uncontrolled catalyst in a regulated metabolism, as cells could not turn it off. In a cytosol full with hundreds of metabolites present in μm to mm concentrations, Ni^0^ would have no choice but to react with whatever reactive moiety came along first. The utility of Ni^0^ in prebiotic biochemical synthesis—capable of performing dozens of different reactions with CO_2_, NH_3_, H_2_ and organic moieties—is its liability in regulated metabolism. The catalytic versatility of Ni^0^ was likely useful in generating organic molecules at origins, but detrimental as enzymatic metabolism reached a state resembling that of modern cells. In order for natural selection in biochemical evolution to take place, enzymes had to be in control of the reactions they catalyzed. That was possible with Ni^2+^ coordinated by N, S, and C in enzymes and cofactors [24] (Fig. 8B,G,H), but not with Ni^0^, which is likely why it was left behind when metabolism escaped the hydrothermal environment [49, 97] within which it arose.

## Material and methods

### Reaction conditions

Each reaction contained 20 mm of pyruvate (Sigma‐Aldrich), or of its respective educt (2‐oxoglutarate, 3‐methyl‐2‐oxopentanoate, 4‐methyl‐2‐oxopentanoate, fumarate; Sigma‐Aldrich); and ammonium chloride when needed, dissolved in HPLC grade water (VWR International, Germany). The pH was adjusted from 7 to 11, depending on the experimental conditions required, by adding 1 M KOH. Ni_2_‐Al_2_/O_3_ (Sigma‐Aldrich) was used as a catalyst, added in a concentration between 0.0 mm to 1.5 mm, depending on the experimental conditions required, per mL of reaction volume, with a final reaction volume of 1.5 mL. Samples were prepared in glass vials (5 mL, Rotilabo‐Rollrändfläschen ND20, Roth) and closed with metal lids (VWR International, Germany). Lids were punctured before being placed inside the reactor (Berghoff BR‐300 with BTC‐3000 temperature controller) to allow for gas exchange. Reactors were filled with 5‐bar argon (99.996%; Messer, Switzerland). Reactions were performed at 25, 40, 60, 80 or 100 °C over 1, 2, 4, 18 or 72 h while stirring at 650 × ***g***.

When the reactions were over, reactors were depressurized and the reaction contents were transferred to 2 mL Eppendorf tubes, and centrifuged for 15 min at 13,000 × ***g*** (Biofuge Fresco, Heraeus) to separate the reaction phases (metal contents and supernatant). The supernatants were then analyzed by NMR.

### Product identification

To prepare the samples for NMR analysis, 600 μL of each sample were transferred into NMR tubes (VWR International, Germany). As a reference for calibration, DSS (2,2‐dimethyl‐2‐silapentane‐5‐sulfonate) was added, reaching a final concentration of 1 mm. NMR spectra were measured on a Bruker Avance III—600 MHz spectrometer by the Center for Molecular and Structural Analytics at Heinrich Heine University Düsseldorf. Spectra were analyzed using Chenomx NMR Suite Version 9.02 software.

## Conflict of interest

The authors declare no conflict of interest.

## Author contributions

CGG, MB, and WFM designed the research. CGG performed the experiments. CGG, MB, and WFM interpreted and analyzed the data. CGG and WFM visualized the data. CGG, MB and WFM wrote the paper. CGG, MB, and WFM edited the paper.

## Supporting information


**Table S1.** Raw data Fig. 1. Raw data of product concentrations with and without nickel and ammonium chloride. Initial concentrations were 20 mm pyruvate and 200 mm ammonium chloride. Ni‐SiO_2_/Al_2_O_3_ (1 mmol of Ni atoms) was added as solid phase powder in a total reaction volume of 1.5 mL. The reaction was performed under a 5 bar Ar atmosphere, initial pH 11 with KOH, the reaction time was 18 h at 100 °C. No H_2_ was added. Reactions were performed in triplicates.
**Table S2.** Raw data Fig. 2. Raw data of product concentrations at different pH values. Initial concentrations were 20 mm of pyruvate. Ni‐SiO_2_/Al_2_O_3_ (1 mmol of Ni atoms) was added as solid phase powder in a total reaction volume of 1.5 mL. The pH was set to 7, 8, 9, 10, and 11 with KOH, respectively, and the reaction time was set to 2 h. The reaction was performed under a 5 bar Ar atmosphere. No H_2_ was added. Each dot represents a single measurement. Dots positioned on the X axis represent measurements where no concentration could be detected. Reactions were performed in triplicates.
**Table S3.** Raw data Fig. 3. Raw data of product concentrations with different temperature and time conditions. Initial concentrations were 20 mm of pyruvate. Ni‐SiO_2_/Al_2_O_3_ (1 mmol of Ni atoms) was added as solid phase powder in a total reaction volume of 1.5 mL. The reaction time was 1 h (B), 2 h (B), 4 h (C), and 18 h (D), respectively. Temperature was 25, 40, 60, 80, and 100 °C. The pH was set to 9 with KOH. The reaction was performed under a 5 bar Ar atmosphere. No H2 was added. Reactions were performed in triplicates.
**Table S4.** Raw data Fig. 4. Raw data of product concentrations with different catalysts. Pyruvate concentration was set to 20 mm. The catalysts (Ni‐SiO_2_/Al_2_O_3_, Nano nickel powder, micro nickel powder, SiO_2_/Al_2_O_3_) were added as 1 mmol of Ni atoms of undissolved solid phase powder in a total reaction volume of 1.5 mL. The reaction was performed at 100 °C, under a 5 bar argon atmosphere, pH was set to 9 with KOH. No H_2_ was added. The reaction time was set to 2 h (A), and 18 h (B). Each reaction was performed in triplicates.
**Table S5.** Raw data Fig. 5. Raw data of product concentrations with different catalyst concentrations. Initial concentrations were 20 mm of pyruvate. Ni‐SiO_2_/Al_2_O_3_ was added as solid phase at a concentration of 0.05, 0.11, 0.22, 0.33, 0.66, 1, and 1.5 mmol of Ni atoms, respectively. The pH was 9 (KOH), and the reaction was performed at 2 h at 100 °C, under a 5 bar Ar atmosphere. No H_2_ was added. Each reaction was performed in triplicates.
**Table S6.** Raw data Fig. 6. Raw data of ketone and double bond reduction product concentrations. Educt concentration was set to 20 mm. Ni‐SiO_2_/Al_2_O_3_ (1 mmol of Ni atoms) was added as solid phase powder in a total reaction volume of 1.5 mL. The reaction time was set to 2 h at 100 °C, and pH was set to 9 with KOH. The reaction was performed under a 5 bar Ar atmosphere. No H_2_ was added. Each reaction was performed in triplicates. (A) Succinate synthesis from fumarate. (B) 2 Hydroxyglutarate synthesis from 2‐oxoglutarate. (C) 2‐Hydroxyisocaproate synthesis from 4‐methyl‐2 oxopentanoate. (D) 2‐Hydroxy‐3‐methylvalerate synthesis from 3‐methyl‐2‐oxopentanoate.
**Table S7.** Raw data Fig. 7. Raw data of reductive amination product concentrations. Initial concentrations were 20 mm of each respective educt and 200 mm ammonium chloride. Ni‐SiO_2_/Al_2_O_3_ (1 mmol of Ni atoms) was added as solid phase powder in a total reaction volume of 1.5 mL. The reaction was performed under a 5 bar Ar atmosphere, initial pH 11 with KOH, the reaction was set at 72 h and 100 °C. No H_2_ was added. Reactions were performed in triplicates. (A) Leucine synthesis from 4‐methyl‐2‐oxopentanoate. (B) Isoleucine synthesis from 3‐methyl‐2‐oxopentanoate. (C) 5‐Oxoproline synthesis from 2‐oxoglutarate.

## Data Availability

The data that supports the findings of this study are available in (Figs 1, 2, 3, 4, 5, 6, 7) and the Supporting Information of this article. Any raw data not specifically shown in the paper will be available upon request.
