# Supplementary material for: Prebiotic aqueous reactions catalyzed by native nickel without hydrogen
Source: FEBS J. Author manuscript; Available in PMC 2026 May 6. (PMC7619042; doi:10.1111/febs.70556)
Supplement: Table S1, Table S2, Table S3, Table S4, Table S5, Table S6, Table S7 [file EMS213496-supplement-Table_S1__Table_S2__Table_S3__Table_S4__Table_S5__Table_S6__Table_S7.pdf]

	Pyruvate [-Ni^0] [-NH_3] (mM)	Pyruvate [-Ni^0] [+NH_3] (mM)	Pyruvate [+Ni^0] [-NH_3] (mM)	Pyruvate [+Ni^0] [+NH_3] (mM)
[1]	15.2761	15.4888	n.d.	n.d.
[2]	14.4107	13.3275	n.d.	n.d.
[3]	17.0135	14.3597	n.d.	n.d.

	Alanine [-Ni^0] [-NH_3] (mM)	Alanine [-Ni^0] [+NH_3] (mM)	Alanine [+Ni^0] [-NH_3] (mM)	Alanine [+Ni^0] [+NH_3] (mM)
[1]	n.d.	n.d.	n.d.	1.4762
[2]	n.d.	n.d.	n.d.	1.2491
[3]	n.d.	n.d.	n.d.	1.1224

	Lactate [-Ni^0] [-NH_3] (mM)	Lactate [-Ni^0] [+NH_3] (mM)	Lactate [+Ni^0] [-NH_3] (mM)	Lactate [+Ni^0] [+NH_3] (mM)
[1]	n.d.	n.d.	9.7135	9.3287
[2]	n.d.	n.d.	10.4968	8.3805
[3]	n.d.	n.d.	9.7113	7.6948

	Acetate [-Ni^0] [-NH_3] (mM)	Acetate [-Ni^0] [+NH_3] (mM)	Acetate [+Ni^0] [-NH_3] (mM)	Acetate [+Ni^0] [+NH_3] (mM)
[1]	0.0148	0.1617	0.8537	0.3320
[2]	n.d.	0.1906	0.8837	0.2974
[3]	0.0554	0.137	0.9038	0.3017

Supplemental Table 1. Raw data Figure 1. Raw data of product concentrations with and without nickel and ammonium chloride. Initial concentrations were 20 mM pyruvate and 200 mM ammonium chloride. Ni-SiO_2/Al_2O_3 (1 mmol of Ni atoms) was added as solid phase powder in a total reaction volume of 1.5 mL. The reaction was performed under a 5 bar Ar atmosphere, initial pH 11 with KOH, the reaction time was 18 h at 100°C. No H_2 was added. Reactions were performed in triplicates.

pH	Pyruvate [1] (mM)	Pyruvate [2] (mM)	Pyruvate [3] (mM)	Lactate [1] (mM)	Lactate [2] (mM)	Lactate [3] (mM)
**7**	n.d.	n.d.	n.d.	11.3001	10.6799	10.8950
**8**	n.d.	n.d.	n.d.	13.4924	16.1508	15.1491
**9**	n.d.	n.d.	n.d.	17.1124	17.4198	17.0749
**10**	n.d.	n.d.	n.d.	14.5391	16.2807	15.7643
**11**	n.d.	n.d.	n.d.	9.7478	10.9123	10.0172

pH	Acetate [1] (mM)	Acetate [2] (mM)	Acetate [3] (mM)
**7**	0.1191	0.0973	0.0879
**8**	0.1281	0.2965	03396
**9**	0.2964	0.4132	0.3113
**10**	0.1296	0.5342	0.7850
**11**	0.2322	0.2650	0.2455

Supplemental Table 2. Raw data Figure 2. Raw data of product concentrations at different pH values. Initial concentrations were 20 mM of pyruvate. Ni-SiO_2/Al_2O_3 (1 mmol of Ni atoms) was added as solid phase powder in a total reaction volume of 1.5 mL. The pH was set to 7, 8, 9, 10, and 11 with KOH, respectively, and the reaction time was set to 2 h. The reaction was performed under a 5 bar Ar atmosphere. No H_2 was added. Each dot represents a single measurement. Dots positioned on the X-axis represent measurements where no concentration could be detected. Reactions were performed in triplicates.

Raw data Figure 3A [1h]

Temperature (°C)	Pyruvate [1] (mM)	Pyruvate [2] (mM)	Pyruvate [3] (mM)	Lactate [1] (mM)	Lactate [2] (mM)	Lactate [3] (mM)
**25**	9.8090	11.0657	11.2670	n.d.	n.d.	n.d.
**40**	13.1949	15.2201	12.0138	n.d.	n.d.	n.d.
**60**	13.4307	15.4054	12.9687	n.d.	n.d.	n.d.
**80**	12.4268	14.1875	11.5273	1.0875	0.7280	1.0111
**100**	0.2891	0.0308	0.0592	14.6500	13.1054	14.0402

Temperature (°C)	Acetate [1] (mM)	Acetate [2] (mM)	Acetate [3] (mM)
**25**	0.0164	0.0155	0.0156
**40**	0.0567	0.0580	0.0497
**60**	0.0917	0.1231	0.0926
**80**	0.1022	0.1334	0.0776
**100**	0.1115	0.0949	0.0835

Raw data Figure 3B [2h]

Temperature (°C)	Pyruvate [1] (mM)	Pyruvate [2] (mM)	Pyruvate [3] (mM)	Lactate [1] (mM)	Lactate [2] (mM)	Lactate [3] (mM)
**25**	14.4576	13.7264	15.4781	n.d.	n.d.	n.d.
**40**	17.0321	14.7814	13.8389	n.d.	n.d.	n.d.
**60**	15.4497	14.9801	15.9499	n.d.	n.d.	n.d.
**80**	1.0500	0.6079	1.1167	12.7927	13.8431	13.9612
**100**	n.d.	n.d.	n.d.	17.1124	17.4198	17.0749

Temperature (°C)	Acetate [1] (mM)	Acetate [2] (mM)	Acetate [3] (mM)
**25**	0.0155	0.0155	0.0230
**40**	0.0529	0.0738	0.0417
**60**	0.0600	0.0553	0.0731
**80**	0.1001	0.1059	0.1116
**100**	0.2964	0.4132	0.3113

Raw data Figure 3C [4h]

Temperature (°C)	Pyruvate [1] (mM)	Pyruvate [2] (mM)	Pyruvate [3] (mM)	Lactate [1] (mM)	Lactate [2] (mM)	Lactate [3] (mM)
**25**	12.0511	11.3594	12.2813	n.d.	n.d.	n.d.
**40**	13.3314	12.6450	12.0363	n.d.	n.d.	n.d.
**60**	10.4015	10.8549	12.3827	1.7391	1.7932	1.8348
**80**	n.d.	n.d.	n.d.	14.4273	12.3039	14.5249
**100**	n.d.	n.d.	n.d.	15.8362	18.0540	15.6835

Temperature (°C)	Acetate [1] (mM)	Acetate [2] (mM)	Acetate [3] (mM)
**25**	0.0226	0.0275	0.0341
**40**	0.1178	0.1007	0.0850
**60**	0.0994	0.0767	0.0947
**80**	0.0478	0.0562	0.0316
**100**	0.1769	0.2044	0.1047

Raw data Figure 3D [18h]

Temperature (°C)	Pyruvate [1] (mM)	Pyruvate [2] (mM)	Pyruvate [3] (mM)	Lactate [1] (mM)	Lactate [2] (mM)	Lactate [3] (mM)
**25**	13.2098	13.7484	12.7345	n.d.	n.d.	n.d.
**40**	13.7183	14.1686	14.3055	n.d.	n.d.	n.d.
**60**	14.3702	11.0668	11.7109	0.3487	0.2389	0.2656
**80**	n.d.	n.d.	n.d.	12.7042	11.7315	11.4080
**100**	n.d.	n.d.	n.d.	11.0396	11.0131	12.1422

Temperature (°C)	Acetate [1] (mM)	Acetate [2] (mM)	Acetate [3] (mM)
**25**	0.0216	0.0010	0.0149
**40**	0.3995	0.4563	0.3244
**60**	0.4048	0.3581	0.3950
**80**	0.2035	0.1508	0.1576
**100**	0.7373	0.8290	0.8542

Supplemental Table 3. Raw data Figure 3. Raw data of product concentrations with different temperature and time conditions. Initial concentrations were 20 mM of pyruvate. Ni-SiO_2/Al_2O_3 (1 mmol of Ni atoms) was added as solid phase powder in a total reaction volume of 1.5 mL. The reaction time was 1h **(B)**, 2h **(B)**, 4h **(C)**, and 18 h **(D)**, respectively. Temperature was 25°C, 40°C, 60°C, 80°C, and 100°C. The pH was set to 9 with KOH. The reaction was performed under a 5 bar Ar atmosphere. No H_2 was added. Reactions were performed in triplicates.

Raw data Figure 4A [2h]

Catalyst	Pyruvate [1] (mM)	Pyruvate [2] (mM)	Pyruvate [3] (mM)	Lactate [1] (mM)	Lactate [2] (mM)	Lactate [3] (mM)
Ni-SiO_2/Al_2O_3	n.d	n.d	n.d	17.8169	17.4659	17.2118
Nano Ni	3.4672	4.0855	3.2751	n.d.	n.d.	n.d.
Micro Ni	15.3600	14.6800	14.5200	n.d.	n.d.	n.d.
SiO_2/Al_2O_3	18.5206	15.8875	14.5200	n.d.	n.d.	n.d.
No catalyst	15.3000			n.d.		

Catalyst	Acetate [1] (mM)	Acetate [2] (mM)	Acetate [3] (mM)
Ni-SiO_2/Al_2O_3	0.2344	0.2488	0.2714
Nano Ni	1.8893	2.0366	1.6803
Micro Ni	0.6600	0.6000	0.6400
SiO_2/Al_2O_3	0.0859	0.0899	0.0885
No catalyst	0.0148		

Raw data Figure 4B [18h]

Catalyst	Pyruvate [1] (mM)	Pyruvate [2] (mM)	Pyruvate [3] (mM)	Lactate [1] (mM)	Lactate [2] (mM)	Lactate [3] (mM)
Ni-SiO_2/Al_2O_3	n.d	n.d	n.d	16.5800	17.4800	17.3100
Nano Ni	4.9600	4.47700	3.1900	6.6800	7.2200	8.3300
Micro Ni	14.6520	15.7367	15.4130	3.0519	3.2605	3.2318
SiO_2/Al_2O_3	19.2806	18.1122	21.2498	n.d.	n.d.	n.d.
No catalyst	17.1400			n.d.		

Catalyst	Acetate [1] (mM)	Acetate [2] (mM)	Acetate [3] (mM)
Ni-SiO_2/Al_2O_3	1.2300	0.7400	0.9500
Nano Ni	2.9000	2.8200	2.7700
Micro Ni	0.4739	0.5534	0.5904
SiO_2/Al_2O_3	n.d.	n.d.	n.d.
No catalyst	0.0554		

Supplemental Table 4. Raw data Figure 4. Raw data of product concentrations with different catalysts. Pyruvate concentration was set to 20 mM. The catalysts (Ni-SiO_2/Al_2O_3, Nano nickel powder, micro nickel powder, SiO_2/Al_2O_3) were added as 1 mmol of Ni atoms of undissolved solid phase powder in a total reaction volume of 1.5 mL. The reaction was performed at 100°C, under a 5 bar argon atmosphere, pH was set to 9 with KOH. No H_2 was added. The reaction time was set to 2 h **(A)**, and 18 h **(B)**. Each reaction was performed in triplicates.

$Ni-SiO_2/Al_2O_3$ (mM)	Pyruvate [1] (mM)	Pyruvate [2] (mM)	Pyruvate [3] (mM)	Lactate [1] (mM)	Lactate [2] (mM)	Lactate [3] (mM)
**0.05**	13.1142	15.3396	12.1251	1.3007	1.1211	1.4426
**0.11**	12.5910	11.9418	10.4562	3.1723	3.3194	3.0858
**0.22**	11.2084	10.7782	11.4425	4.2738	4.2992	4.9393
**0.33**	7.5484	7.8566	9.5385	6.0045	7.4879	6.9266
**0.66**	1.4955	1.7374	2.4170	14.9149	14.2723	12.5128
**1**	n.d.	n.d.	n.d.	17.4198	17.0749	17.1124
**1.5**	n.d.	n.d.	n.d.	17.6590	16.0863	16.5755

$Ni-SiO_2/Al_2O_3$ (mM)	Acetate [1] (mM)	Acetate [2] (mM)	Acetate [3] (mM)
**0.05**	0.5562	0.8460	0.6070
**0.11**	0.1510	0.1342	0.0922
**0.22**	0.1483	0.1821	0.2157
**0.33**	0.1348	0.1464	0.1534
**0.66**	0.1731	0.1403	0.1361
**1**	0.4132	0.3113	0.2964
**1.5**	0.1464	0.1467	0.1197

Supplemental Table 5. Raw data Figure 5. Raw data of product concentrations with different catalyst concentrations. Initial concentrations were 20 mM of pyruvate. $Ni-SiO_2/Al_2O_3$ was added as solid phase at a concentration of 0.05, 0.11, 0.22, 0.33, 0.66, 1, and 1.5 mmol of Ni atoms, respectively. The pH was 9 (KOH), and the reaction was performed at 2 h at 100°C, under a 5 bar Ar atmosphere. No H_2 was added. Each reaction was performed in triplicates.

Raw data Figure 6A

	Fumarate (mM)	Succinate (mM)
**[1]**	n.d.	24.2600
**[2]**	n.d.	24.3900
**[3]**	n.d.	24.7000

Raw data Figure 6B

	2-Oxoglutarate (mM)	2-Hydroxyglutarate (mM)
**[1]**	n.d.	21.7220
**[2]**	n.d.	20.1870
**[3]**	n.d.	20.3880

Raw data Figure 6C

	4-Methyl-2-oxopentanoate (mM)	2-Hydroxyisocaproate (mM)
**[1]**	0.2931	19.5348
**[2]**	0.7288	19.5591
**[3]**	0.7584	19.3958

Raw data Figure 6D

	3-Methyl-2-oxopentanoate (mM)	2-Hydroxy-3-methylvalerate (mM)
**[1]**	0.9678	12.4926
**[2]**	0.8970	13.4035
**[3]**	0.6670	12.6702

Supplemental Table 6. Raw data Figure 6. Raw data of ketone and double bond reduction product concentrations. Educt concentration was set to 20 mM. Ni-SiO_2/Al_2O_3 (1 mmol of Ni atoms) was added as solid phase powder in a total reaction volume of 1.5 mL. The reaction time was set to 2 h at 100°C, and pH was set to 9 with KOH. The reaction was performed under a 5 bar Ar atmosphere. No H_2 was added. Each reaction was performed in triplicates. **A.** Succinate synthesis from fumarate. **B.** 2-Hydroxyglutarate synthesis from 2-oxoglutarate. **C.** 2-Hydroxyisocaproate synthesis from 4-methyl-2-oxopentanoate. **D.** 2-Hydroxy-3-methylvalerate synthesis from 3-methyl-2-oxopentanoate.

Raw data Figure 7A

	4-Methyl-2-oxopentanoate (mM)	Leucine (mM)	2-Hydroxyisocaproate (mM)
[1]	n.d.	1.3336	11.6780
[2]	n.d.	2.2125	11.8850
[3]	n.d.	1.3001	12.1904

Raw data Figure 7B

	3-Methyl-2-oxopentanoate (mM)	Isoleucine (mM)	2-Hydroxy-3-methylvalerate (mM)
[1]	n.d.	0.9117	8.0438
[2]	n.d.	1.2431	7.5011
[3]	n.d.	0.7421	6.4658

Raw data Figure 7C

	2-Oxoglutarate (mM)	2-Hydroxyglutarate (mM)	Glutamate (mM)	2-Oxoproline (mM)
[1]	n.d.	1.7629	n.d.	11.8812
[2]	n.d.	2.6339	n.d.	11.4198
[3]	n.d.	1.4145	n.d.	11.1556

Supplemental Table 7. Raw data Figure 7. Raw data of reductive amination product concentrations. Initial concentrations were 20 mM of each respective educt and 200 mM ammonium chloride. Ni-SiO_2/Al_2O_3 (1 mmol of Ni atoms) was added as solid phase powder in a total reaction volume of 1.5 mL. The reaction was performed under a 5 bar Ar atmosphere, initial pH 11 with KOH, the reaction was set at 72 h and 100°C. No H_2 was added. Reactions were performed in triplicates. **A.** Leucine synthesis from 4-methyl-2-oxopentanoate. **B.** Isoleucine synthesis from 3-methyl-2-oxopentanoate. **C.** 5-Oxoproline synthesis from 2-oxoglutarate.
